# Dietary intake and micronutrient adequacy among adults engaged in agriculture in rural Sri Lanka: findings from a cross-sectional baseline survey

**DOI:** 10.1017/S1368980025000072

**Published:** 2025-01-30

**Authors:** Caroline A Joyce, Bess L Caswell, Aulo Gelli, Sonja Y Hess, Hasara Sitisekara, Christine P Stewart, Xiuping Tan, Renuka Jayatissa, Kalana Peiris, Renuka Silva, Deanna K Olney

**Affiliations:** 1 University of California Davis, Department of Nutrition, 1 Shields Ave, Davis, CA 95616-5270, USA; 2 United States Department of Agriculture, Agricultural Research Service, Western Human Nutrition Research Center, John E. Thurman, Jr. Laboratory, 620 W Health Science Dr, Davis, CA 95616, USA; 3 International Food Policy Research Institute, 1201 I St NW, Washington, DC 20005, USA; 4 Wayamba University of Sri Lanka, Department of Applied Nutrition, Faculty of Livestock, Fisheries & Nutrition, Wayamba University of Sri Lanka, Makandura, Gonawila, North Western Province, LK 60170, Sri Lanka; 5 Medical Research Institute Sri Lanka, Dr. Danister De Silva Mawatha (Baseline road), Colombo 08, Sri Lanka; 6 World Food Programme, No: 2 Jawatte Ave, Colombo 00500, Sri Lanka

**Keywords:** Diet assessment, Prevalence of adequacy, Micronutrient intake, Fruit and vegetables

## Abstract

**Objective:**

To characterise food group consumption, assess the contribution of food groups to energy and micronutrient intake, and estimate usual nutrient intake among adults in rural Sri Lanka.

**Design:**

A baseline survey (December 2020–February 2021) was conducted as part of an agriculture-based, nutrition-sensitive resilience program evaluation. Dietary intake was assessed using telephone-based 24-h recalls (*n* 1283), with repeat recalls from 769 participants. Mean daily intake of food groups and their contribution to energy and nutrient intakes were calculated. The National Cancer Institute method was used to estimate usual intakes and the prevalence of adequate micronutrient intake (PAI). Differences by sex, district, and wealth were assessed using *t* tests and ANOVA.

**Setting:**

Forty-five rural villages throughout Sri Lanka.

**Participants:**

Men and women from households in the program evaluation study area.

**Results:**

On average, grains and coconut milk provided 56 % and 12 % of energy, respectively. Rice, fish, dairy, and pulses were the primary sources of micronutrients. Participants consumed 118 ± 117 g of vegetables and 71 ± 243 g of fruit per day. PAI was < 25 % for calcium, zinc, niacin, folate, and vitamins B_6_, B_12_, and C, reflecting low consumption of animal-source foods (80 g/day), whole grains, fruit, and vegetables (F&V). Significant differences in food group consumption by socio-demographic subgroup were observed among districts and wealth quintiles.

**Conclusions:**

We observed high consumption of rice and coconut milk and low prevalence of micronutrient adequacy. We recommend increasing animal-source food, whole grain, and F&V consumption to close nutrient gaps, as well as research to identify effective solutions to increase micronutrient intake.

Healthy diets are critical for preventing disability and chronic diseases and for optimal human functioning across the lifespan. Fruit and vegetables (F&V) are particularly important for protection against cancer, metabolic diseases and cardiovascular disease^(1)^. Individual-level dietary data are needed to better understand food group consumption and micronutrient intake in low- and middle-income countries.

In 2021, the Sri Lanka Ministry of Health published a revision to the national food-based dietary guidelines (FBDG), which recommend that half of the diet come from unrefined grains, cereals, and starchy staples; one-third from vegetables; and the remainder from protein-rich foods^(2)^. The guidelines also recommend that adults consume ≥ 3 servings of vegetables and ≥ 2 servings of fruit per day. Despite this guidance, the typical diet in Sri Lanka includes high consumption of cereals and low consumption of micronutrient-dense foods, including meat, eggs, nuts, seeds, dairy, dark leafy greens, and deep orange F&V^(3,4)^. Rice is the leading source of calories, and only 25 % of Sri Lankan adults consume at least the recommended 5 F&V servings/day, which places individuals at risk of micronutrient deficiency^(3–5)^. Animal-source food intake is also low in Sri Lanka, which may be due in part to cultural and religious preferences towards plant-based diets^(6)^. Based on 2013 food balance sheet estimates, at least one third of Sri Lankans were at risk of inadequate folate, zinc, and vitamin B_12_ intake, and nearly the entire population was at risk of inadequate calcium and riboflavin intake^(7)^. While previous research offers some understanding of food group consumption among Sri Lankans, statistically rigorous evidence on nutrient intake and the prevalence of adequate micronutrient intake is lacking, particularly among women outside of childbearing years and men of any age^(3–5)^.

National estimates from 2022 suggest that 8·2 % and 18·5 % of Sri Lankan men and women experienced anaemia, respectively, while 2·5 % and 7·2 % were iron deficient^(8)^. Nationally representative data regarding other micronutrient deficiencies in Sri Lanka are lacking. In addition to micronutrient deficiencies, the country is undergoing a transition towards energy-dense, nutrient-poor, ultra-processed foods, increasing the risk of obesity and chronic diseases^(9–11)^. The prevalence of obesity and overweight is estimated to have doubled between 2000 and 2019 – from 18 % to 30 % among women and from 11 % to 20 % among men^(11)^. The prevalence of diabetes among adults increased nearly 60 % in the same period, and although women remain more likely to experience diabetes, the prevalence among men has increased more rapidly^(11)^.

While under- and over-nutrition threaten the health of Sri Lankan adults, a recent review found that the cost of a diet meeting the national FBDG exceeds household food expenditures for more than one-third of households^(12)^. Moreover, Sri Lanka has witnessed an increase in the incidence and intensity of climate shocks in recent decades, including intense droughts, rains, landslides and floods^(13)^. In a country where nearly 85 % of food is produced domestically, these shocks significantly increase the vulnerability of farmers and consumers to food insecurity^(13,14)^.

The aim of the present study was to provide evidence about the dietary intake of rural Sri Lankan adults to inform the development of sustainable and cost-effective interventions for improving nutrition and health outcomes via improved diet quality. In particular, we targeted adults engaged in agriculture – the predominant livelihood activity in Sri Lanka – living in districts which are highly vulnerable to climate shocks and food and nutrition insecurity. We sought to identify the specific types of F&V already consumed by the study population which could be promoted in future interventions and government programmes to improve nutrient intakes. The specific objectives of this analysis were to characterise food group consumption, to estimate observed and usual nutrient intakes and the adequacy of micronutrient intakes, and to examine the relative contribution of F&V to micronutrient intake.

## Methods

This study is a secondary analysis of data from a longitudinal evaluation of the Resilience, Risk Reduction, Recovery, Reconstruction, and Nutrition (R5N) program, which sought to assess impacts of the R5N Food Assistance for Assets nutrition-sensitive agriculture and resilience program, with and without a health promotion component. The evaluation was carried out by the International Food Policy Research Institute (IFPRI), in collaboration with the World Food Program (WFP), Wayamba University of Sri Lanka, Medical Research Institute Sri Lanka, and the University of California, Davis.

### Study population

**Figure 1.**
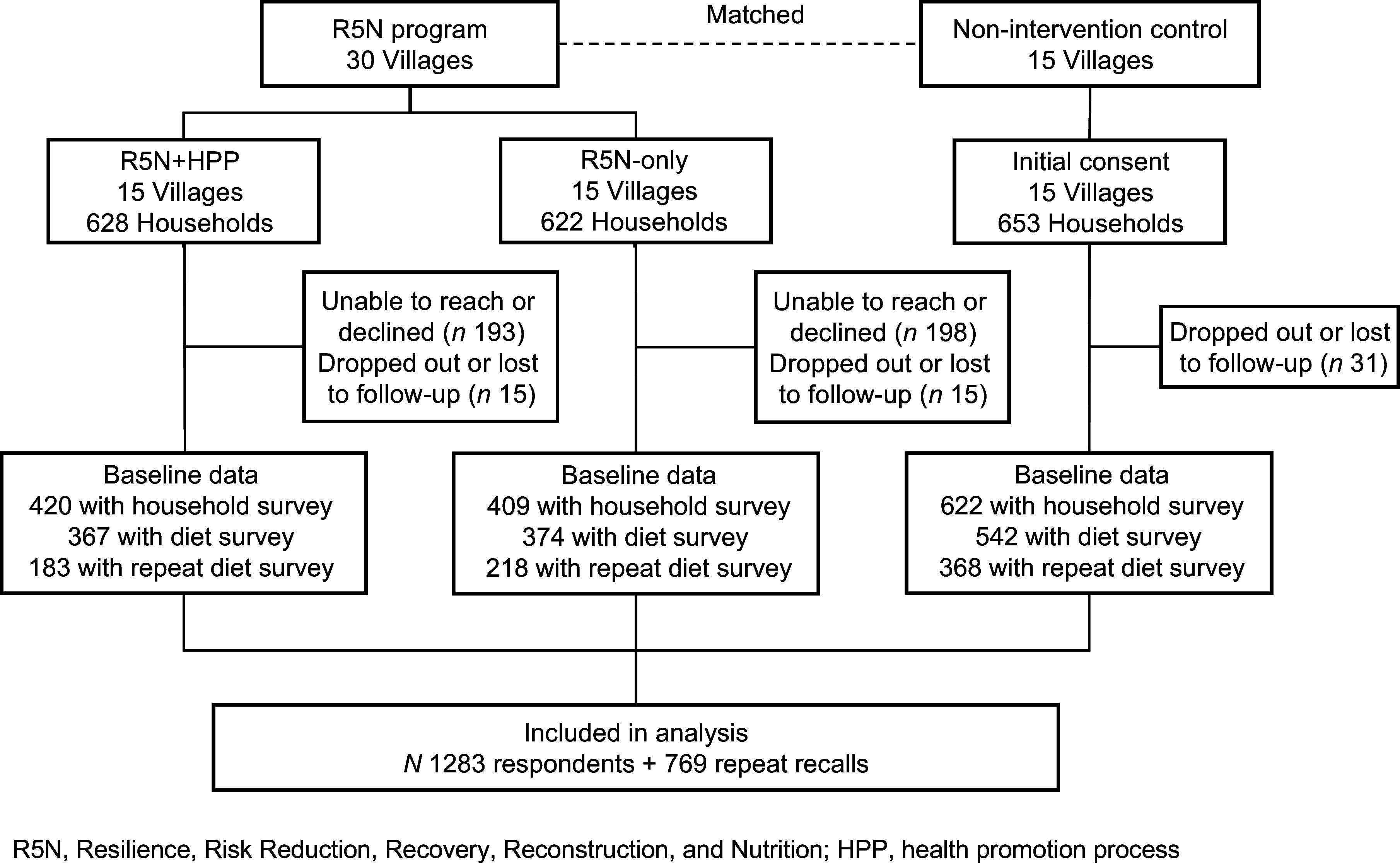
CONSORT flow diagram of participants through the R5N evaluation study

The target population was adults (≥ 18 years old) living in five rural agricultural districts across Sri Lanka. The program villages were selected due to their vulnerability to environmental shocks and nutrient insecurity. Thirty villages were selected to receive the R5N program by WFP. The program evaluation team randomly selected half of the R5N villages to receive the health promotion component and fifteen additional control villages from the same districts based on a community-level matching procedure. The matching variables included demographic factors, precipitation, temperature, land cover, soil characteristics, proximity to cities, and nighttime light density. Households were eligible for inclusion if at least one adult member of the household had engaged in farming or livestock rearing in the past year. In the control group villages, the research team used stratified random sampling to enroll households for which phone numbers could be obtained from electoral lists. The study team sought to enroll all R5N beneficiaries and obtained their phone numbers from WFP’s beneficiary list. Additional details regarding the R5N sample selection and methodology are available elsewhere^(15)^. For the R5N evaluation study, the research team collected data from the primary R5N beneficiary (in the intervention arms) or the household member who was most involved in agriculture (in the control group). If this person was unwilling or unable to participate, another adult member of the household was asked to complete the dietary survey. Figure 1 illustrates the flow of participants through the study. The final sample size of the baseline dietary survey was 1283 adults. Assuming 30 % prevalence of adequate intake (PAI), a sample size of 1283 provides a precision of ±2·5 % in estimating PAI with 95 % confidence. If PAI is 50 %, our precision is ±2·7 %.

### Data collection

Baseline data were collected from December 2020 to February 2021. It included three tablet-assisted phone calls to collect information regarding household characteristics and assets, nutrition knowledge, food security, participation in agriculture, and R5N program exposure. Additional socio-demographic information was collected using a household survey. Variables included sex, age, educational attainment, household income and household expenditure.

Dietary data were obtained using a 24-h recall survey conducted via telephone due to the onset of the COVID-19 pandemic and the need to reduce in-person contact. Data were entered into electronic forms in SurveyCTO using tablets^(16)^. Interviews were conducted using the multiple-pass method, with respondents reporting all foods and beverages consumed on the previous day^(17)^. Participants reported portion sizes using a pre-determined list of household utensils that are commonly used in Sri Lanka (e.g. tea cups and coconut serving spoons). Repeat recalls were collected from a random subgroup of the sample on a non-consecutive day. Although information on supplement use was collected, the data were not incorporated into the analysis. Only 4 % of respondents reported using a specific micronutrient supplement, and the quantity with which they supplemented was not known.

### Data analysis

We used Stata version 17 for data cleaning, management and descriptive analyses^(18)^. The wealth index was calculated using principal component analysis of twenty-four household assets (e.g. material of dwelling floor, access to electricity, and television ownership).

To estimate the quantity of each food reported, we applied standardised gram weight conversion factors based on food type and portion unit. When mixed dishes were reported, we disaggregated them using ingredient fractions from a standard recipe database developed by the study team from prior dietary surveys, local informants, and searches of published Sri Lankan recipes. We developed a recipe calculator to estimate the nutritional composition of mixed dishes using ingredient-level nutrient data from the Sri Lankan food composition table^(19)^. Food composition table data were supplemented with values from neighboring countries or the United States Department of Agriculture (USDA) when necessary. The calculator also incorporated nutrient retention factors for cooking and yield factors (to account for water gains or losses) from the USDA^(20,21)^. Each ingredient was assigned a nutrient retention factor based on the assigned food sub-group (e.g. type of meat, type of vegetable, etc.) and primary cooking method of the mixed dish (e.g. boiled, sauteed, fried). The yield factors and ingredient fractions were applied and refined using an iterative process. The Wayamba University research team had previously estimated the nutrient values for nearly 400 recipes using the Foodbase 2000 software (Institute of Brain Chemistry, UK). After validating our recipe calculator output against these values, we were able to estimate the yield factors of similar recipes for which we did not have pre-existing nutrient values. The resulting ingredient-level dataset, including foods reported ‘as consumed’ (e.g. fruit, snacks, and beverages), served as the basis for the food group analysis.

We assessed consumption of individual foods/ingredients using two levels of food group classification and one additional classification of only F&V. The broadest level included fourteen food groups, which were modified from the FAO individual dietary diversity score (Table 1)^(22)^. In comparison to the individual dietary diversity score, the present analysis combined meat and eggs due to the low frequency and quantity of consumption in our study population. In contrast, pulses were separated from nuts and seeds since the local diet includes regular consumption of pulses. The remaining reported food items fell into three distinct categories (beverages, coconut milk, and spices/seasonings) and were categorised as such, rather than ‘miscellaneous’, as used in the individual dietary diversity score, due to the important contribution of coconut milk and seasonings to micronutrient intake in our study population. In this analysis, we sought to gain a deeper understanding of the contribution of F&V to nutrient adequacy and therefore used a further classification of twenty-five food groups which disaggregated F&V (Table 1). Categorisation was based on standard food groups and nutrient profiles. The food groups were refined during data analysis to include only categories which contributed ≥ 2 %, on average, to total intake of one or more micronutrients. For the final analysis, we assessed the proportional contribution of each reported F&V to the total pool of micronutrients obtained only from F&V. As above, we refined the categories during analysis and reported those which contributed ≥ 2 % of one or more micronutrients. Based on this approach, we retained sixteen unique F&V and seven F&V groups (Table 1). We performed descriptive statistics characterising the quantities of foods consumed and the contribution of each food group to total nutrient intake in Stata^(19)^. A serving of F&V was defined as 80 g^(23)^. We used ANOVA tests and Jonckheere–Terpstra tests for trend to assess whether the proportion of energy derived from each food group differed by demographic subgroup (sex, district, and wealth quintile).

**Table 1.** Definition of the three levels of food group categorization used to assess consumption among rural Sri Lankan adults, with comparison to the food groups used to calculate the FAO individual dietary diversity score^(23)^

FAO Individual dietary diversity score (IDDS)	Group 1	Group 2	Group 3
Cereals	Grains	Refined grains	
		Whole grains	
Sugar/honey	Sweets	Sweets	
Milk/ milk products	Dairy	Dairy	
Meat, poultry, offal	Meat and eggs	Meat	
Eggs		Eggs	
Fish and seafood	Fish and shellfish	Fish and shellfish	
Oil/fats	Oils and fats	Oils and fats	
Pulses/ legumes/ nuts	Pulses	Pulses	
	Nuts and seeds	Nuts and seeds	
Root and tubers	Starchy roots and tubers	Starchy roots and tubers	
Miscellaneous	Coconut milk	Coconut milk	
	Beverages	Beverages	
	Spices and seasonings	Spices and seasonings	
			Curry leaves
Fruits	Fruits	Bananas	Bananas
		Mango	Mango
		Other fruit	Nelli
			Papaya
			Other fruits
Vegetables	Vegetables	Cabbage	Cabbage
		Capsicum and chilies	Capsicum and chilies
		Dark leafy greens	Amaranth leaves
			Cassava leaves
			Drumstick leaves
			Hummingbird tree leaves
			Sessile joyweed
			Water spinach
			Other dark leafy greens
		Green and winged beans	Green and winged beans
		Gourds	Pumpkin
			Other gourds
		Other vegetables	Eggplant
			Other vegetables
		Root vegetables	Carrot
			Onion
			Other root vegetable

We estimated the usual intake distributions of macro- and micronutrients using the National Cancer Institute method in SAS version 9.4^(24,25)^. We also estimated the PAI for eleven micronutrients (calcium (expressed in mg), iron (mg), zinc(mg), vitamin A (µg of retinol equivalents; RE), thiamine (mg), riboflavin (mg), niacin (mg), vitamin B_6_ (mg), folate (µg dietary folate equivalents; DFE), vitamin B_12_ (µg), and vitamin C (mg)). Vitamin A was expressed in RE to align with The European Food Safety Authority’s population reference intake and the harmonised average nutrient requirements (H-ARs)^(26,27)^. To estimate the 95 % CI, we used bootstrap resampling and calculated standard errors from the bootstrapped estimates. PAI was calculated as the proportion of the study sample whose intake exceeded the age- and sex-specific H-AR, which were developed for applicability at the global level^(27)^. For iron and zinc, we selected the requirements associated with moderate absorption and semi-unrefined diets, respectively. For iron, the H-AR assumes 10 % absorption due to moderate phytate intake and some consumption of meat and fish. Similarly for zinc, the increased requirement accounts for moderate phytate intake (assuming 900 mg/person/day)^(27)^.

Nine micronutrients (calcium, zinc, vitamin A, thiamine, riboflavin, niacin, vitamin B_6_, folate, and vitamin C) were analysed using the Simulating Intake of Micronutrients for Policy Learning and Engagement (SIMPLE) SAS macro, which reduces the processing time required to run the National Cancer Institute method^(28)^. Vitamin B_12_ was consumed episodically, i.e. > 10 % of the study sample did not consume it on the recall day. Therefore, we used the two-part National Cancer Institute model to estimate the probability of consumption and the consumption-day amount^(29)^. Finally, to assess the PAI of iron, we used the SIMPLE-Iron SAS macro, assuming a mixture of oral contraceptive users and non-users in the population^(30)^. The analysis of iron is unique since the distribution of requirements for menstruating women does not satisfy the assumption that nutrient requirements are symmetrical and thus requires use of the full-probability method of analysis^(30)^. PAI was analysed by sex, wealth quintile, and district. We used *t* tests to assess whether differences in the PAI of micronutrients differed by demographic subgroup. Batticaloa and wealth quintile 1 were used as the reference groups for hypothesis testing between districts and wealth quintiles, respectively.

## Results

The study sample included 1283 individuals, with repeated 24-h recalls from 769 participants (60 %) collected 3–10 d after the initial interview. Approximately one-third of respondents were female (*n* 486), and the median age was 44 years, ranging from 18 to 88 (Table 2). Enrollment by district as a percentage of the total sample ranged from 13 % in Mannar in the north to 25 % in Monaragala in the south.

**Table 2.** Baseline characteristics of rural Sri Lankan adults (*n* 1283) in the resilience, risk reduction, recovery, reconstruction and nutrition (R5N) evaluation study (2020–2021)

Demographic characteristic	*n*	%
Age, years		
Mean	45·1	
sd	12·6	
Sex, female	486	38·6
District		
Batticaloa	265	20·7
Mannar	172	13·4
Matale	217	16·9
Monaragala	318	24·8
Mullaitivu	311	24·2
Schooling, head of household, years		
No schooling	73	5·8
1–4	223	17·7
5–8	413	32·8
9–12	542	43·0
> 12	10	0·8
Household size, number of occupants		
Mean	4.2	
sd	1.6	

### Food group consumption

**Figure 2.**
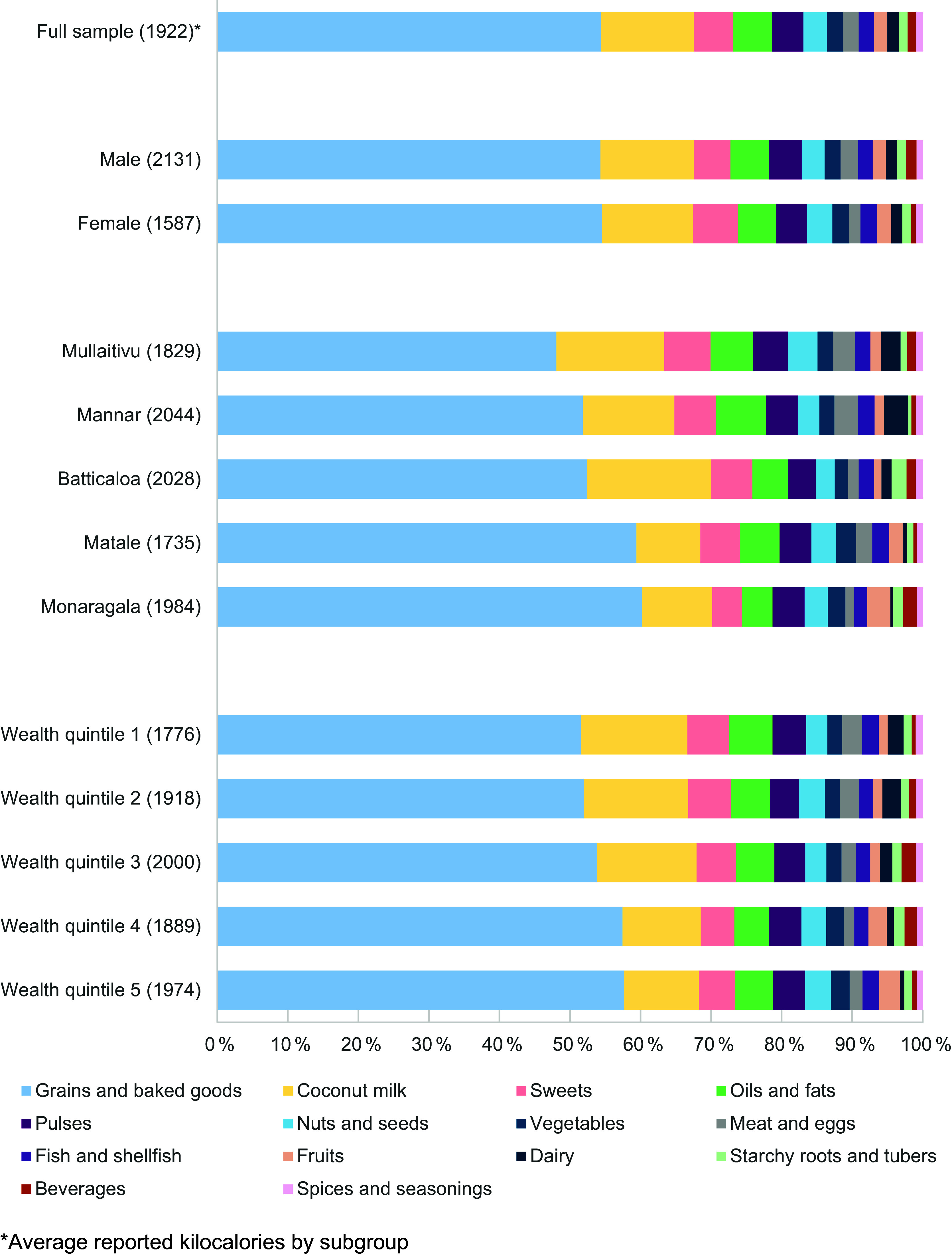
Average daily contribution of 14 food groups to total energy intake among rural Sri Lankan adults (%, *n* 1283)

Average daily reported energy intake was 1922 ± 836 kcal (Figure 2). More than half of energy came from grains, including baked goods and snacks (56 %). Coconut milk contributed 12 % of reported energy, followed by sweets and added sugars (6 %). Together, these food groups accounted for nearly three quarters of the average diet. White rice alone (including parboiled, polished, and flour varieties) supplied 29 % of energy intake, while F&V contributed just 4 % of energy, on average.

As a proportion of total energy, male and female food group consumption was similar (Figure 2). Three food groups were significantly different by sex: meat and eggs (2·2 ± 5·0 % of kcal among men *v*. 1·5 ± 3·4 % among women, *P* < 0·01), sweets (5·4 ± 6·0 % of kcal *v*. 6·1 ± 6·7 %, respectively, *P* = 0·04), and spices/seasonings (0·8 ± 0·5 % of kcal *v*. 0·9 ± 0·7 %, respectively, *P* = 0·01).

There was no difference in the proportion of energy from pulses, seafood, beverages, or spices/seasonings by geographic region (*P* > 0·05). All other food group differences were statistically significant (all *P* < 0·01). Participants in Matale and Monaragala, the central and southernmost study sites, respectively, reported higher proportions of energy from grains, fruit, and vegetables and lower proportions of energy from sweets, dairy, and coconut milk relative to participants in the other three districts. Participants in Mannar and Mullaitivu, the northernmost study districts, reported the highest consumption of sweets, oils/fats, meat/eggs, and dairy and the lowest proportion of energy from grains.

Differences in the proportion of energy from grains, coconut milk, fruit, vegetables, and dairy were statistically significant between wealth quintiles. As wealth quintile increased, the proportional consumption of grains and vegetables decreased, and consumption of coconut milk and dairy increased (*P* < 0·01). Although the test for trend in fruit consumption was not statistically significant across wealth quintiles (*P* = 0·1), the percent of energy derived from fruit was nearly twice as high among participants in the lowest two quintiles compared to those in the highest three quintiles, on average (2·4 ± 5·7 % of kcal *v*. 1·3 ± 3·0 %, *P* < 0·01). Similarly, the test for trend was NS for meat and egg consumption, but participants in the highest three wealth quintiles derived a higher proportion of energy from meat and eggs than those in the lowest two wealth quintiles (2·2 ± 4·8 % of kcal *v*. 1·6 ± 3·8 %, *P* = 0·02).

### Usual nutrient intake and prevalence of adequate micronutrient intake

The estimated usual energy intake in the study sample was 1836 ± 22 kcal (Table 2). Usual carbohydrate, fat, and protein intakes were 292 ± 3 g, 56 ± 1 g, and 53 ± 1 g, respectively. Of the total kilocalories consumed, 62 ± 10 % was derived from carbohydrates, 26 ± 9 % from fat, and 11 ± 10 % from protein. Alcohol contributed an additional < 1 ± 4 %.

PAI was low for most of the micronutrients examined in this analysis (Table 3). PAI was highest for thiamine (68 %) and riboflavin (64 %). High thiamine intake was largely due to parboiled rice (22 % of total intake), while parboiled and red rice, milk powder, and brewed black tea contributed the largest proportions of riboflavin (5–9 % each). PAI was modest for iron (34 %) and vitamin A RE (33 %). For all other nutrients, the prevalence was below 25 %. Dietary intakes of calcium, vitamin C, and zinc were the most problematic, with 3 %, 8 %, and 8 % of the sample consuming adequate amounts, respectively.

**Table 3.** Estimated usual nutrient intakes and the prevalence of adequate intake (PAI) of micronutrients among rural Sri Lankan adults (*n* 1283)

	Mean	95 % CI	Median	PAI, %	95 % CI
Energy (kcal)	1836·4	1793·1, 1879·7	1744·3	n/a	
Carbohydrates (g)	292·1	285·8, 298·4	278·6	n/a	
Fat (g)	56·4	54·4, 58·4	51·9	n/a	
Protein (g)	53·1	51·4, 54·7	49·7	n/a	
Calcium (mg)	353·7	339, 368·4	319·9	3·0	1·8, 4·2
Iron (mg)	10·0	9·6, 10·4	9·3	34·2	31·3, 37
Zinc (mg)	6·2	5·9, 6·4	5·7	8·4	6·5, 10·3
Vitamin A (µg RE)	501·7	431·3, 572·2	396·5	33·0	25·7, 40·3
Thiamine (mg)	2·1	1·7, 2·4	1·4	67·9	61·2, 74·5
Riboflavin (mg)	2·7	2·3, 3·1	1·8	63·6	57·6, 69·5
Niacin (mg)	8·3	8, 8·7	7·6	18·5	15·5, 21·5
Vitamin B_6_ (mg)	1·1	1, 1·2	0·9	23·9	18·9, 29
Folate (µg DFE)	200·5	190·1, 210·8	174·9	24·7	21·4, 28·1
Vitamin B_12_ (µg)	1·1	1, 1·2	0·9	12·3	8·7, 15·8
Vitamin C (mg)	42·8	39·3, 46·4	35·1	7·8	4·7, 10·9

**Figure 3.**
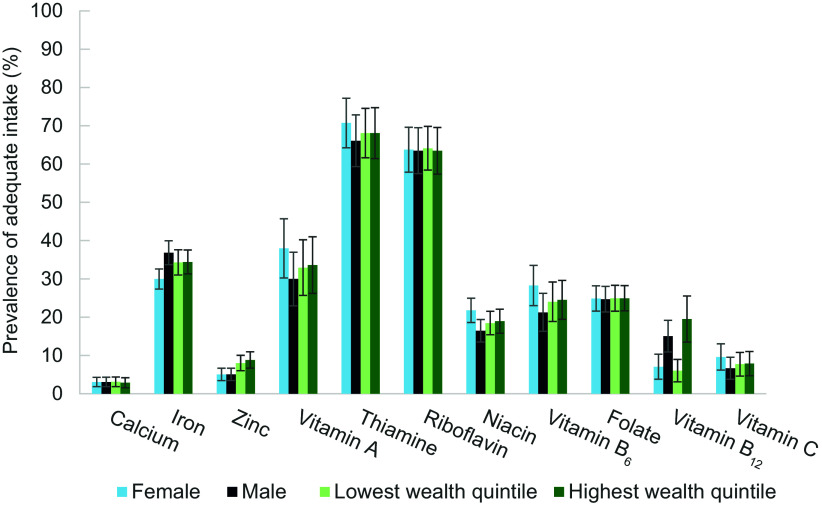
Prevalence of adequate intake of 11 key micronutrients among rural Sri Lankan adults by sex and wealth quintile (%, *n* 1283)

The prevalence of adequate iron, zinc, and niacin intakes was statistically significantly different between men and women: 30 % of women consumed adequate iron *v*. 37 % of men (*P* < 0·01), 14 % of women consumed adequate zinc *v*. 5 % of men (*P* < 0·01), and 22 % of women consumed adequate niacin *v*. 16 % of men (all *P* < 0·01) (Figure 3). There was little variation in PAI among the sampled districts or wealth quintiles. Vitamin B_12_ was the only nutrient for which the PAI was statistically significantly different across districts and wealth quintiles. It ranged from 16 % adequate in Batticaloa to 9 % in Mullaitivu (*P* = 0·03), and it was negatively correlated with wealth quintile, ranging from 20 % among the wealthiest participants to 6 % among the poorest (*P* < 0·01).

### Average contribution of food groups to micronutrient intake

**Figure 4.**
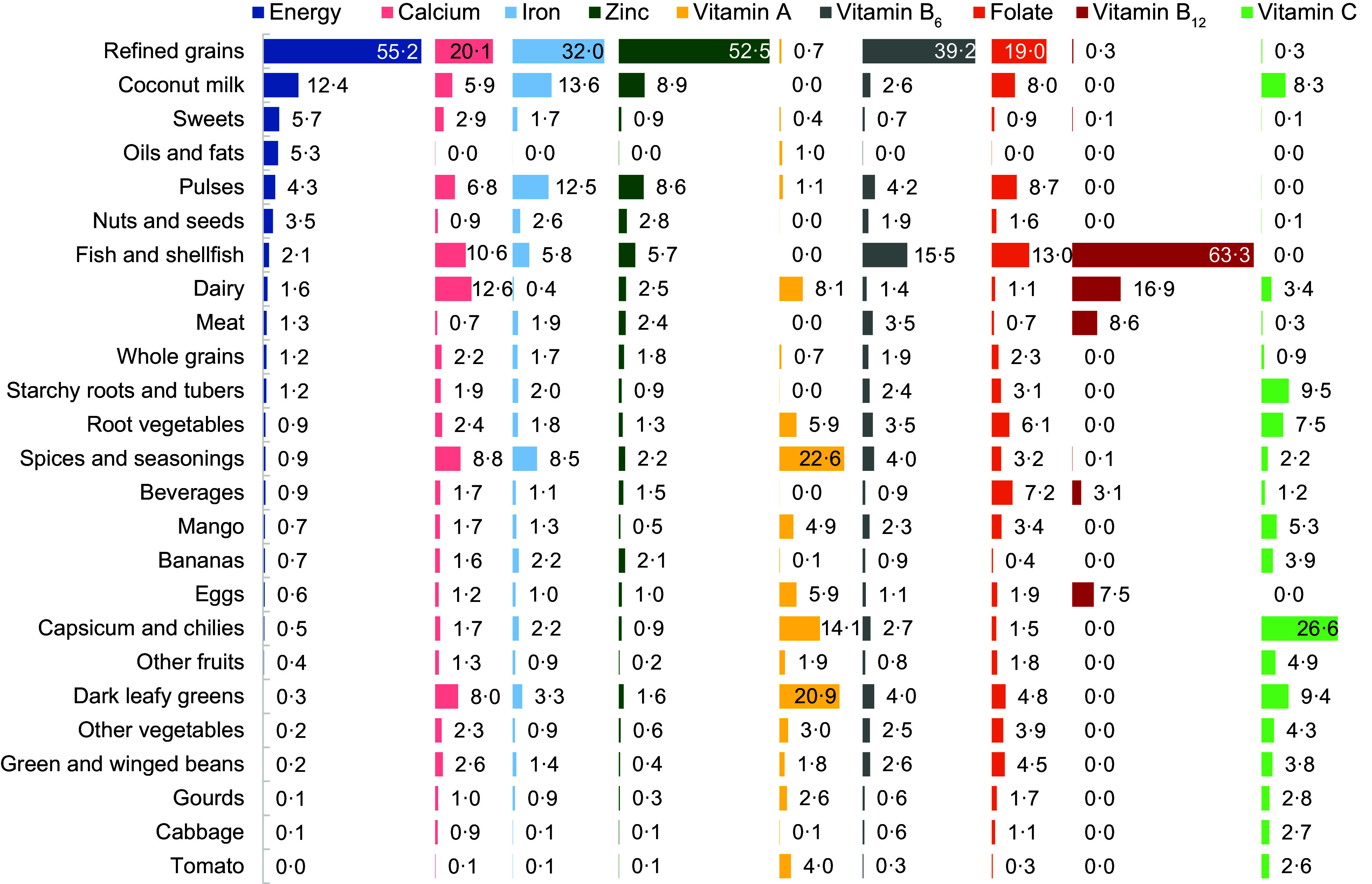
Relative contribution of 25 food groups to energy and micronutrient intakes among rural Sri Lankan adults (%, *n* 1283)

Refined grains, including baked goods and snacks, provided the largest proportions of calcium, iron, zinc, thiamine, riboflavin, niacin, vitamin B_6_, and folate (19–55 % of reported intake per person, on average) due to the large quantities consumed (Figure 4).

The study population consumed a variety of more nutrient-dense foods, including fish/shellfish, dairy, pulses, and capsicum/chilies. Fish and shellfish contributed nearly two-thirds of the sample’s vitamin B_12_ intake and 11–16 % of calcium, vitamin B_6_, and folate (Figure 4). Capsicums and chilies were the largest contributor of vitamin C (26 %) and the third largest source of vitamin A RE (14 %). Spices/seasonings (primarily curry leaves) and dark leafy greens supplied the highest proportions of vitamin A RE (23 % and 21 %, respectively). Other notable sources of micronutrients include dairy (the second largest source of vitamin B_12_ and calcium), pulses (the third highest contributor of iron and thiamine), and coconut milk (the second largest source of iron).

### Relative contribution of individual fruit and vegetables to total micronutrient intake from all F&V

Reported consumption of fruit was 71 ± 243 g, or 0·9 ± 3·0 servings, per day and vegetables was 118 ± 117 g, or 1·5 ± 1·5 servings, per day. Out of the nutrients that participants obtained from F&V, mango accounted for the highest proportions of vitamin C, folate, vitamin A RE, and vitamin B_6_ (18–39 %) intake; the second highest proportions of thiamine and riboflavin intake; and the third highest proportions of iron and calcium (8–16 %) (see online supplementary material, Supplemental Figure 1). Capsicum and chilies supplied the largest proportions of thiamine, riboflavin, and niacin, and the second largest proportions of F&V-specific vitamin C, iron, and vitamin B_6_ intake. Bananas were the most significant F&V source of zinc and iron (30 % and 15 %, respectively), while green/winged beans and curry leaves provided the greatest proportions of niacin and calcium from F&V, respectively (26 % and 13 %).

## Discussion

On average, respondents derived 56 % of their total energy from grains, more than half of which was from white rice. Despite being a relatively poor source of micronutrients, refined grains provided the largest proportions of eight key micronutrients due to the large quantities consumed. Of the eleven micronutrients assessed, the PAI for seven nutrients was < 25 %. Approximately one-third of the study sample reported adequate intake of iron and vitamin A. PAI was highest for riboflavin and thiamine (approximately 65 %), reflecting overall low micronutrient intake. The average consumption of F&V in our sample was 189 g, or 2·4 ± 3·5 servings, per day. Out of the nutrients that participants obtained from F&V, mango, capsicum/chilies, bananas, green/winged beans, and curry leaves were the most significant sources of micronutrients.

The Sri Lankan FBDG recommend that adults consume at least 400 g/day of F&V (3–5 servings of vegetables and 2–3 servings of fruit)^(2)^. In the present study, participants consumed less than half of the minimum recommendation. A nationally representative survey conducted in Sri Lanka in 2021 reported F&V consumption of 4·6 servings per day among adults (1·2 and 3·3 servings of F&V, respectively), which is nearly double the amount reported in our study population^(5)^. One might expect that the higher intake of F&V in the national sample was due to the inclusion of individuals with higher income; however, we found lower F&V consumption among the highest wealth quintiles in our study population. COVID-19 likely affected consumption of F&V and animal-source foods among our sample. Throughout 2020, Sri Lankan consumers were affected by market closures, significant disruptions in the local supply chain, loss of livelihoods, and reduced access to animal feed^(31)^. Although the government distributed seed packets and promoted home gardens nationwide, the uptake and establishment of the national home gardening program is not well understood. Moreover, newly established crops or gardens may not have yielded edible F&V for several months after distribution. Differing data collection methods likely also played a role. The national survey used a food frequency questionnaire (FFQ) to estimate the average numbers of servings consumed per day from self-reported consumption days per week and the number of servings per consumption-day^(5)^. Furthermore, the F&V serving sizes were depicted on cards shown to the participants, which do not necessarily align with the 80 g/serving conversion used in the present analysis.

In an earlier nationally representative survey in Sri Lanka, which collected data in 2011 using 24-h recalls, adults reported consuming 2·2 servings/day of F&V^(4)^. The slightly lower consumption of F&V reported by Jayawardena *et al.* relative to our findings is consistent with the trend of increasing F&V consumption over time seen in the recurring STEPwise approach to non-communicable diseases risk factor surveillance (STEPS) surveys in Sri Lanka^(5)^. In the 2006 STEPS survey, respondents reported consuming 3·2 servings/day of F&V, which increased to 4·3 servings/day in the 2015 survey, and 4·6 in the aforementioned 2021 survey. Additionally, the 2011 survey by Jayawardena *et al.* reported that males consumed more F&V than females (2·4 *v*. 2·0 servings/day)^(4)^. This may partially explain the higher intake in our study, given the overrepresentation of males.

While the contribution of total energy from protein in our study sample was similar to a nationally representative study conducted in Sri Lanka in 2014, the contribution from fat and carbohydrates diverged^(32)^. In our sample, 26 % of energy came from fat and 62 % from carbohydrates, *v*. 19 % and 71 %, respectively, in the study conducted by Jayawardena, *et al.* ^(32)^ These differences may be partially due to the use of in-person recalls and portion size estimation aids in the 2014 study. Moreover, the data reported by Jayawardena *et al.* were collected 10 years prior to the data presented in the current analysis. Due to significant evolution of the political landscape and increased income levels in Sri Lanka in the past half century, food consumption patterns have shifted noticeably^(10,33)^. The nutrition transition may have contributed to the higher proportion of fat in our sample. In the present study, biscuits contributed the largest proportion of fat intake (> 7 %) after coconut milk, which illustrates a shift towards ultra-processed packaged foods. The findings from a more recent national survey, which collected data in late 2021, were consistent with our results. The mean energy intake in the survey by Jayatissa *et al.* was 1902 kilocalories, 63 % of which came from carbohydrates, 22 % from fat and 12 % from protein^(34)^. Participants in their study reported slightly higher mean energy and macronutrient intakes, which may be due to the inclusion of respondents in urban areas and/or with a higher socio-economic status, sampling respondents several months after the largest waves of COVID-19 had passed, and the collection of data at the household level (from which individual-level intake was estimated using consumption units)^(35)^.

A 2020 USDA commodity report stated that annual per capita rice consumption in Sri Lanka was 107 kg, which amounts to 293 g/day^(36)^. This figure is relatively consistent with our findings, in which respondents consumed 235 g/day of rice (all varieties combined). The overestimation by the USDA is likely explained by the use of Household Consumption and Expenditure Survey data, which does not account for spoilage or food waste at the household level^(37)^. The participants in our study reported approximately 13 servings/day of grains and starchy staples, which is the maximum recommended by the national FBDG (8–13 servings)^(2)^. Although the guidelines recommend that grains be primarily whole or unrefined, nearly all of the grains and starchy staples reported by our respondents were refined. A recent literature review found that red rice and other traditional varieties are re-gaining popularity in Sri Lanka, but supply has not yet caught up with increasing demand, leading to high retail prices and limited affordability for consumers^(10)^.

Coconut milk was the second largest source of energy and the largest source of fat in our sample, which aligns with findings from a recent FBDG technical review^(38)^. In the present study, participants reported 103 g/day of coconut milk – slightly higher than the 90 g/day estimated in a 2019 24-h recall survey among Sri Lankan women^(38)^. Again, the difference is likely due to the inclusion of men in our study, who reported > 30 % higher energy intake than women and the same proportion of energy from coconut milk.

Study participants living in Matale and Monaragala reported the lowest proportions of energy intake from dairy, coconut milk, and sweets and the highest proportions of energy from grains, fruit, and vegetables. These districts are predominantly Sinhala, whose traditional diet is largely comprised of rice with small portions of 1–2 vegetables. In Mannar and Mullaitivu, participants reported the highest consumption of meat, eggs, and dairy, which could be partly due to traditional dietary customs typical for those districts. Wealth distribution within the study sample likely also accounts for some of the differences in food group consumption, as participants living in Matale and Monaragala were more likely to be in the lowest two wealth quintiles compared to respondents in the other three study areas. The trends we observed with respect to wealth quintile (i.e. a positive correlation with animal-source foods and negative correlation with grains, fruit, and vegetables) are consistent with the global nutrition transition^(33)^.

The aforementioned 2019 survey of Sri Lankan women reported that cereals, green leafy vegetables, and pulses were the top three contributors to iron intake, and that vegetables, fruit, eggs, and dairy were the top four contributors to vitamin A intake^(38)^. In our study sample, cereals were also the largest contributor to iron intake, followed by coconut milk and pulses. Our results for vitamin A were also similar – vegetables were the primary contributor, followed by spices/seasonings (i.e. curry leaves), dairy, fruit, and eggs. In terms of specific F&V, both studies found mango to be the leading source of vitamin A.

Among our study participants, seafood was an important contributor of vitamin B_6_, vitamin B_12_, calcium, and folate. Per capita consumption was 32 g/day, which is on par with the national average of 37 g/person/day estimated in 2021 by the Sri Lankan Ministry of Fisheries^(39)^. Despite the contribution of seafood to adequate micronutrient intake in our study sample, overall intake of the micronutrients that seafood can provide was concerningly low (i.e. calcium, iron, and zinc). To some extent, this may be related to the decline in fish production in Sri Lanka in recent years as a result of the COVID-19 pandemic and economic crisis, which lead to fuel shortages^(40)^. In addition to seafood, increasing consumption of pulses, dairy, and eggs – foods that are already consumed in modest quantities in the study population – could help close some of the gaps between reported and recommended micronutrient intakes.

For both men and women, the usual intake of carbohydrates in our sample was 292 g, and the usual protein intake was 53 g. Carbohydrate consumption was more than double the 130 g recommended daily allowance for adults, and although protein intake was sufficient for women (recommended daily allowance = 46 g), it was below the 56 g recommended daily allowance for men^(30)^. To our knowledge, no studies have published estimates using statistically modelled usual micronutrient intakes or PAI based on usual intake among Sri Lankan adults. A recent nationally representative study in Sri Lanka assessed micronutrient intake by estimating intake from household-level consumption data^(34)^. The median reported intakes for calcium, iron, niacin, and vitamin B_6_ were similar to our findings (±15 %); however, they found significantly lower intakes of vitamin A, thiamine, riboflavin, folate, and vitamin C and significantly higher median intakes of zinc and vitamin B_12_. Concordantly, the PAI for zinc and vitamin B_12_ estimated by Jayatissa *et al.* were considerably higher than the PAI in our sample. The divergence in results may be due to differing analytical approaches. Because of the high degree of within-person variation in dietary intake, the removal of this variation through measurement error modelling is important for obtaining unbiased estimates of the PAI^(24)^. Moreover, the higher observed intakes of zinc and vitamin B_12_ in the nationally representative sample may be due to the inclusion of urban populations who have higher incomes, on average, and therefore consume larger quantities of animal-source foods.

Recent studies in Cambodia and South India estimated PAI using similar statistical methods to ours, and their findings suggest that some variation in micronutrient intake exists among different adult population groups in South Asia^(41,42)^. Of the six micronutrients assessed in the Cambodia analysis, the PAI for four nutrients was within 10 % of our results (calcium, zinc, vitamin A, and riboflavin). They found a higher PAI for iron (50 % compared to 34 % among our sample) and a lower PAI for thiamine (46 % *v*. 68 %, respectively)^(41)^. The higher overall consumption of food, and thus micronutrients, among men in our study may explain some of the incongruity, since the study in Cambodia was conducted only among rural-dwelling women.

Among the sample in India, the PAI was again similar for zinc and vitamin A, in addition to vitamin B_12_, and the PAI for iron was substantially higher (89 %)^(42)^. There was less discrepancy in the PAI for thiamine, but the prevalence in India was still lower by 10 % (58 % *v*. 68 % in our sample). In contrast, the study in India found substantially higher PAI for calcium, niacin, and vitamin C and lower PAI for riboflavin and folate. Of note, the analysis by Shalini *et al.* averaged three 24-h recalls to estimate usual nutrient intake before calculating the probability of adequacy relative to requirements. As noted previously, not accounting for within-person variation may have biased their estimates^(24)^. Nevertheless, the differences in PAI appear to be at least partially explained by actual differences in food consumption. For example, Shalini *et al.* found that milk/ milk products contributed 60 % of calcium in their sample compared with 13 % in rural Sri Lanka. Similarly, fruit, green leafy vegetables, and pulses contributed a substantially larger proportion of vitamin C, iron, and folate among South Indian respondents compared with our respondents^(42)^.

In the demographic subgroup comparison of PAI, we found statistically significant differences between men and women for iron, zinc, and niacin. It is worth noting, however, that the estimated usual intakes of these nutrients were the same for men and women and that the difference in PAI was a result of different H-ARs between the sexes. We also found a statistically significant correlation between the PAI of vitamin B_12_ and wealth quintile, which is consistent with our findings that dairy, meat, and egg consumption increased with wealth quintile. In low- and middle-income countries, these food groups are relatively expensive compared to starchy staples^(43)^. In one study, fish was the most expensive food group per 1000 kcal; however, it is the most important source of B_12_ in our study population, and we found no difference in the proportion of energy from seafood by wealth quintile^(43)^. This suggests that promoting fish intake could be an equitable way to achieve adequate B_12_ intake in our study population.

There are several limitations to consider in the present analysis. Recall bias is an inherent source of error in retrospective self-reported data collection. However, this bias tends to be lower for 24-h recall, since it only requires the participant to recount intake from the previous day, while other types of dietary data collection methods (e.g. FFQ) use a longer recall period^(44)^. The onset of the COVID-19 pandemic and necessity of conducting interviews via telephone instead of in-person may have decreased the precision of portion size estimations because food models and portion size tools could not be shown during the interview. Participants were asked to report portion sizes using household utensils and dishware that are common in Sri Lanka, although those same utensils may not have been present in every respondent’s home. During protocol modifications, the study team dedicated significant effort into developing the list of possible portion sizes for each food item to reduce reporting error.

Few studies have compared the validity of phone-based 24-h recalls relative to those collected in-person. The only study to our knowledge which included a gold standard reference value reported that both recall methods underestimated true energy intake, but that the mean phone and in-person energy intake estimates were not different from each other^(45)^. Another study, which had a larger sample including men and women, found no difference in reported mean energy or protein intakes between phone and in-person interviews^(46)^. Given the uncertainty around the performance of phone-based 24-h recalls, we developed a follow-up study to validate phone and in-person recalls against weighed food records. Preliminary results show that, within our study population, phone recalls performed as well as those conducted in-person across several metrics^(47)^. Therefore, we expect that the use of phone-based interviews did not have a significant impact on the validity of our findings.

Although the use of telephone-based interviews may have introduced selection bias, given the ubiquity of telephone subscriptions in Sri Lanka, it is unlikely that the use of phone surveys had a significant impact on the generalisability of our findings^(48)^. There is some evidence from a subset of adult women in another rural district in Sri Lanka that more than 98 % had at least one mobile phone in the household^(49)^. In fact, the use of phone surveys may have decreased selection bias since we were able to survey participants who work long hours outside the home and may not have been available for in-person surveys. Nevertheless, the present study was not intended to be regionally or nationally representative. The objective of the study was to assess the food and nutrient intake of a population that is particularly vulnerable to climate shocks and – consequently – food and nutrient insecurity. As a result, we cannot necessarily extrapolate these results to all rural-dwelling adults in Sri Lanka.

Finally, we recognise that the findings from our study may not reflect typical nutrient intakes in Sri Lanka due to COVID-19, political and economic turmoil in the country, and increasing frequency of climate shocks. In particular, the pandemic affected global supply chains and the availability of some food commodities, as well as national revenue from tourism, which may have affected consumption patterns during the data collection period. However, the R5N evaluation study collected data at four time points throughout 2021–2024, which will provide additional information on food group consumption and micronutrient intake in the same population after the country had recovered from COVID-19 and the economic crisis. The follow-up data will enable us to assess the extent to which these external factors may have played a role in changing food consumption in Sri Lanka.

Strengths of our study include rigorous enumerator training, a large sample size, quantitative dietary intake estimates, the compilation of a recipe database and dietary reference tables specific to Sri Lanka, and comprehensive data cleaning activities. One of the most important elements of our study was the collection of repeat recalls from more than half of respondents and use of the National Cancer Institute method for estimating usual nutrient intake, which accounts for intra-individual variation in food consumption.

### Conclusions

The goal of our analysis was to assess food group consumption, nutrient intakes, adequacy of micronutrient intakes relative to requirements, and the contribution of specific F&V to micronutrient intake. Our findings are consistent with previous studies that have observed dietary quality and diversity in low- and middle-income countries well below what is recommended for optimal health. Estimated usual intakes of calcium, vitamin C, zinc, vitamin B_12_, and niacin were particularly low in our study population. The micronutrient gaps are driven by high consumption of rice and low consumption of F&V and animal-source foods.

F&V are a critical source of fibre, micronutrients, and phytonutrients in the diet, and inadequate consumption has adverse consequences on one’s risk of cardiovascular disease, cancer, and all-cause mortality^(1)^. A 2015 survey estimated that > 90 % of Sri Lankan adults have at least one risk factor for non-communicable diseases, and that > 70 % of the disease burden in the country is due to non-communicable diseases^(5)^. Improving consumption of these food groups is imperative to improving health outcomes in the country. Cost, availability, personal preferences, social and cultural norms, workload and time pressures, and environmental factors have been reported as significant barriers to adequate F&V consumption in low- and middle-income countries^(50)^.

More research is needed to identify and evaluate solutions to increasing intake of micronutrient-rich foods such as F&V and animal-source foods in the study population. For example, nutrition-sensitive agricultural interventions may be used to introduce more drought-resilient, nutrient dense crop varieties or to diversify livelihoods. The agricultural sector in Sri Lanka may also benefit from the introduction of technologies to reduce reliance on manual labor and to better predict forthcoming climate shocks. While this survey provides useful insight into the nutrient intakes of a nutritionally vulnerable population, we recommend a future assessment of usual nutrient intakes among a nationally representative sample of adults in Sri Lanka. While increasing F&V consumption would close some micronutrient gaps in the population in a way that is environmentally sustainable and affordable for consumers, improving accessibility of animal-source foods and/or food fortification will likely also be needed to effectively increase the PAI of certain micronutrients (e.g. calcium and zinc).

## Supporting information

Joyce et al. supplementary material Joyce et al. supplementary material

## Supplementary material

For supplementary material accompanying this paper visit https://doi.org/10.1017/S1368980025000072

## References

[1] Aune D , Giovannucci E , Boffetta P et al. (2017) Fruit and vegetable intake and the risk of cardiovascular disease, total cancer and all-cause mortality-a systematic review and dose-response meta-analysis of prospective studies. Int J Epidemiol 46, 1029–1056. 28338764 10.1093/ije/dyw319 PMC5837313

[2] Ministry of Health, Nutrition Division (2021) Food Based Dietary Guidelines for Sri Lankans: Practitioner’s Handbook, Report no. 3rd ed. Sri Jayawardenepura Kotte: Ministry of Health, Nutrition Division.

[3] Weerasekara PC , Withanachchi CR , Ginigaddara GAS et al. (2020) Understanding dietary diversity, dietary practices and changes in food patterns in marginalised societies in Sri Lanka. Foods 9, E1659. 10.3390/foods9111659 PMC7696452 33202762

[4] Jayawardena R , Byrne NM , Soares MJ et al. (2013) Food consumption of Sri Lankan adults: an appraisal of serving characteristics. Public Health Nutr 16, 653–658. 22784794 10.1017/S1368980012003011 PMC10271271

[5] WHO (2024) STEPwise Approach to NCD Risk Factor Surveillance (STEPS): Sri Lanka (Internet). World Health Organization (WHO). https://www.who.int/teams/noncommunicable-diseases/surveillance/data/sri-lanka (accessed 30 March 2023).

[6] Abeywickrema S , Gunathunga S , Walpita JK et al. (2024) Evaluating sensory impacts of sustained plant-based diets: altered sensitivity and hedonic responses to meat-related odours in Sri Lankan young adults. Food Qual Preference 117, 105151. doi: 10.1016/j.foodqual.2024.105151

[7] Mark HE , Houghton LA , Gibson RS et al. (2016) Estimating dietary micronutrient supply and the prevalence of inadequate intakes from national Food Balance Sheets in the South Asia region. Asia Pac J Clin Nutr 25, 368–376. doi: 10.6133/apjcn.2016.25.2.119 27222421

[8] Jayatissa R , Perera A & Alwis N (2023) National Nutrition and Micronutrient Survey in Sri Lanka: 2022. Colombo, Sri Lanka: Department of Nutrition, Medical Research Institute in partnership with UNICEF and WFP.

[9] Seferidi P , Hone T , Duran AC et al. (2022) Global inequalities in the double burden of malnutrition and associations with globalisation: a multilevel analysis of Demographic and Health Surveys from 55 low-income and middle-income countries, 1992–2018. Lancet Global Health 10, e482–90. 35148831 10.1016/S2214-109X(21)00594-5 PMC8924053

[10] Bandara S , Kumara T , Dharmadasa S et al. (2021) Changes in food consumption patterns in Sri Lanka: food security and sustainability: a review of literature. Open J Social Sci 9, 213–237.

[11] Global Nutrition Report (2022) Country Nutrition Profiles: Sri Lanka (Internet). https://globalnutritionreport.org/resources/nutrition-profiles/asia/southern-asia/sri-lanka/ (accessed 10 October 2022).

[12] Dizon F , Herforth A & Wang Z (2019) The cost of a nutritious diet in Afghanistan, Bangladesh, Pakistan, and Sri Lanka. Global Food Secur 21, 38–51.

[13] Gunaratne MS , Radin Firdaus RB & Rathnasooriya SI (2021) Climate change and food security in Sri Lanka: towards food sovereignty. Humanit Soc Sci Commun 8, 1–14.38617731

[14] Institute of Policy Studies (2018) Climate Change, Food Security and Rural Livelihoods in Sri Lanka. Colombo, Sri Lanka: Institute of Policy Studies.

[15] Singh N , Scott S , Kumar N et al. (2023) Food insecurity and perceived effects of COVID-19 on livelihoods in Rural Sri Lanka. Food Nutr Bull 44, 229–239. 37700715 10.1177/03795721231197249 PMC10725086

[16] SurveyCTO (2019) (Internet) Massachusetts, USA: Dobility, Inc. https://www.surveycto.com (accessed March 2023).

[17] Gibson R & Ferguson E (1999) An Interactive 24-Hour recall for Assessing the Adequacy of Iron and Zinc intakes in Developing Countries. Washington, DC: ILSI Press.

[18] StataCorp LLC (2021) Stata Statistical Software: Release 17. College Station, TX: StataCorp LLC.

[19] Jayatissa R , Perera AG , Silva BG et al. (2021) Sri Lanka Food Composition Tables. Colombo, Sri Lanka: Medical Research Institute of Sri Lanka.

[20] USDA (2007) Table of Nutrient Retention Factors: Release 6 (Internet). Maryland, USA: U.S. Department of Agriculture (USDA). 2007 Dec; available at https://www.ars.usda.gov/arsuserfiles/80400530/pdf/retn06.pdf (accessed April 2023).

[21] USDA (1975) Food Yields Summarized by Different Methods of Preparation (Internet). Washington, DC: U.S. Department of Agriculture (USDA). 1975 Sep; available at https://www.ars.usda.gov/ARSUserFiles/80400530/pdf/ah102.pdf (accessed April 2023).

[22] Kennedy G , Ballard T & Dop M (2010) Guidelines for Measuring Household and Individual Dietary Diversity (Internet). Rome: Food and Agriculture Organization of the United Nations (FAO); available at https://www.fao.org/3/i1983e/i1983e.pdf (accessed 31 March 2023).

[23] WHO STEPS (2017) Surveillance Manual (Internet). Geneva, Switzerland: World Health Organization (WHO); available at https://www.who.int/docs/default-source/ncds/ncd-surveillance/steps/steps-manual.pdf (accessed 29 March 2024).

[24] Tooze JA , Kipnis V , Buckman DW et al. (2010) A mixed-effects model approach for estimating the distribution of usual intake of nutrients: the NCI method. Stat Med 29, 2857–2868. doi: 10.1002/sim.4063. 20862656 PMC3865776

[25] SAS Institute Inc. (2023) SAS/STAT® User’s Guide, Version 9.4. Cary, NC: SAS Institute Inc.

[26] EFSA Panel on Dietetic Products, Nutrition, and Allergies (NDA) (2015) Scientific opinion on dietary reference values for vitamin A. EFSA J 13, 4028. doi: 10.2903/j.efsa.2015.4028 PMC13151645 42109809

[27] Allen LH , Carriquiry AL & Murphy SP (2020) Perspective: proposed harmonized nutrient reference values for populations. Adv Nutr 11, 469–483. 31701998 10.1093/advances/nmz096 PMC7231601

[28] Luo H , Dodd KW , Arnold CD et al. (2021) Introduction to the SIMPLE macro, a tool to increase the accessibility of 24-hour dietary recall analysis and modeling. J Nutr 151, 1329–1340. 33693802 10.1093/jn/nxaa440 PMC8112768

[29] Tooze JA , Midthune D , Dodd KW et al. (2006) A new statistical method for estimating the usual intake of episodically consumed foods with application to their distribution. J Am Diet Assoc 106, 1575–1587. 17000190 10.1016/j.jada.2006.07.003 PMC2517157

[30] Institute of Medicine (US) (2000) Standing Committee on the Scientific Evaluation of Dietary Reference Intakes. DRI Dietary Reference Intakes: Applications in Dietary Assessment (Internet), pp. 73–105. Washington, DC: National Academies Press (US); available at https://ods.od.nih.gov/HealthInformation/nutrientrecommendations.aspx (accessed 19 July 2022).

[31] van Buitenlandse ZM (2020) Impact of COVID-19 on Food Supply Chains in Sri Lanka. Agricultural News Abroad. May 27, 2020. https://www.agroberichtenbuitenland.nl/actueel/nieuws/2020/05/27/impact-of-covid19-on-food-supply-chains-in-sri-lanka (accessed 09 September 2024).

[32] Jayawardena R , Thennakoon S , Byrne N et al. (2014) Energy and nutrient intakes among Sri Lankan adults. Int Arch Med 7, 34. 25067954 10.1186/1755-7682-7-34 PMC4110527

[33] Weerahewa J , Sewwandi Wijetunga C , Chandra Babu S et al. (2018) Food Policies and Nutrition Transition in Sri Lanka: Historical Trends, Political Regimes, and Options for Interventions. Washington, DC: International Food Policy Research Institute.

[34] Jayatissa R , Jayawardana R , Perera AG et al. (2023) Nutritional status and dietary intake of the population aged 1–60 years during the COVID-19 Pandemic in Sri Lanka. Ceylon Med J 68, 9–20. 37609911 10.4038/cmj.v68iSI1.9749

[35] Wickramasinghe D & Fernando VK (2022) Sri Lanka’s fight against COVID-19: a brief overview. In: Pandemic Risk, Response, and Resilience: COVID-19 Responses in Cities Around the World. 1st ed. pp. 129–42. Boston, MA: Elsevier.

[36] Galappattige A (2020) Grain and Feed Annual (Internet). New Delhi: U.S. Department of Agriculture (USDA). 2020 May. Report No.: CE2020–0005; available at =https://apps.fas.usda.gov/newgainapi/api/Report/DownloadReportByFileName?fileName=Grain%20and%20Feed%20Annual_New%20Delhi_Sri%20Lanka_03-27-2020 (accessed April 2024).

[37] Fiedler JL , Lividini K , Bermudez OI et al. (2012) Household Consumption and Expenditures Surveys (HCES): a primer for food and nutrition analysts in low- and middle-income countries. Food Nutr Bull 33, S170–184. 23193768 10.1177/15648265120333S205

[38] Technical Review Report (2021) 2019–2020 Sri Lankan Food Based Dietary Guide Lines Evidence Review (Internet). Sri Lanka: Ministry of Health, Nutrition and Indigenous Medicine (MoH) Sri Lanka; available at https://nutrition.health.gov.lk/wp-content/uploads/2021/10/Tec-Report-Final-draft.pdf (accessed 03 April 2024).

[39] State Ministry of Ornamental Fish, Inland Fish & Prawn Farming, Fishery Harbour Development, Multiday Fishing Activities and Fish Exports (2021) Annual Performance Report for the Year 2021 (Internet). Colombo, Sri Lanka: State Ministry of Ornamental Fish, Inland Fish & Prawn Farming, Fishery Harbour Development, Multiday Fishing Activities and Fish Exports. Report no. 405; available at https://www.parliament.lk/uploads/documents/paperspresented/1657004026002914.pdf (accessed 03 April 2024).

[40] National Aquatic Resources Research and Development Agency (NARA) (2023) *Sri Lanka Fisheries Industry Outlook 2022*. http://www.nara.ac.lk/wp-content/uploads/2023/10/Fisheries-Industry-Outlook-2022.pdf (accessed April 2024).

[41] Verbowski V , Talukder Z , Hou K et al. (2018) Effect of enhanced homestead food production and aquaculture on dietary intakes of women and children in rural Cambodia: a cluster randomized controlled trial. Matern Child Nutr 14, e12581. 29314705 10.1111/mcn.12581 PMC6866186

[42] Shalini T , Sivaprasad M , Balakrishna N et al. (2019) Micronutrient intakes and status assessed by probability approach among the urban adult population of Hyderabad city in South India. Eur J Nutr 58, 3147–3159. 30511165 10.1007/s00394-018-1859-y

[43] Bai Y , Alemu R , Block SA et al. (2021) Cost and affordability of nutritious diets at retail prices: evidence from 177 countries. Food Policy 99, 101983. 33767525 10.1016/j.foodpol.2020.101983 PMC7970354

[44] International Dietary Data Expansion Project (2025) 24-hour Dietary Recall (24HR) (Internet). https://inddex.nutrition.tufts.edu/data4diets/data-source/24-hour-dietary-recall-24hr (accessed 10 June 2023).

[45] Tran KM , Johnson RK , Soultanakis RP et al. (2000) In-person *v.* telephone-administered multiple-pass 24-hour recalls in women: validation with doubly labeled water. J Am Diet Assoc 100, 777–783. doi: 10.1016/S0002-8223(00)00227-3 10916515

[46] Bogle M , Stuff J , Davis L et al. (2001) Validity of a telephone-administered 24-hour dietary recall in telephone and non-telephone households in the rural Lower Mississippi Delta region. J Am Diet Assoc 101, 216–222. doi: 10.1016/S0002-8223(01)00056-6 11271695

[47] Joyce CA , Stewart CP , Arnold CD et al. (2025) Relative validity of interviewer-administered 24-hour recalls collected by telephone and in-person versus weighed food records amongrural Sri Lankan adults. Unpublished manuscript. 10.1016/j.cdnut.2026.107672 PMC13091110 42005786

[48] World Bank Open Data (2023) Mobile Cellular Subscriptions (per 100 people) - Sri Lanka (Internet). https://data.worldbank.org (accessed 24 May 2023).

[49] Jayasinghe I , Wickramasinghe Y , Kurera DM et al. (2022) Feasibility of using telephone interviews and internet-based message services during the COVID-19 pandemic in rural Sri Lanka: experiences of the Rajarata Pregnancy Cohort. Rural Remote Health 22, 7442. doi: 10.22605/RRH7442 35546146

[50] Kehoe SH , Dhurde V , Bhaise S et al. (2019) Barriers and facilitators to fruit and vegetable consumption among rural Indian women of reproductive age. Food Nutr Bull 40, 87–98. 30974984 10.1177/0379572118816459 PMC6660308

