# Supplementary material for: Dietary intake and micronutrient adequacy among adults engaged in agriculture in rural Sri Lanka: findings from a cross-sectional baseline survey
Source: Public Health Nutr. 2025 Jan 30;28(1):e35. doi: 10.1017/S1368980025000072 (PMC11984007; doi:10.1017/S1368980025000072)

Supplemental Figure 1. Relative contribution of specific fruits and vegetables to total micronutrient intake from fruits and vegetables among Sri Lankan farming adults (% of total nutrients supplied by fruit & vegetables, N=1283)

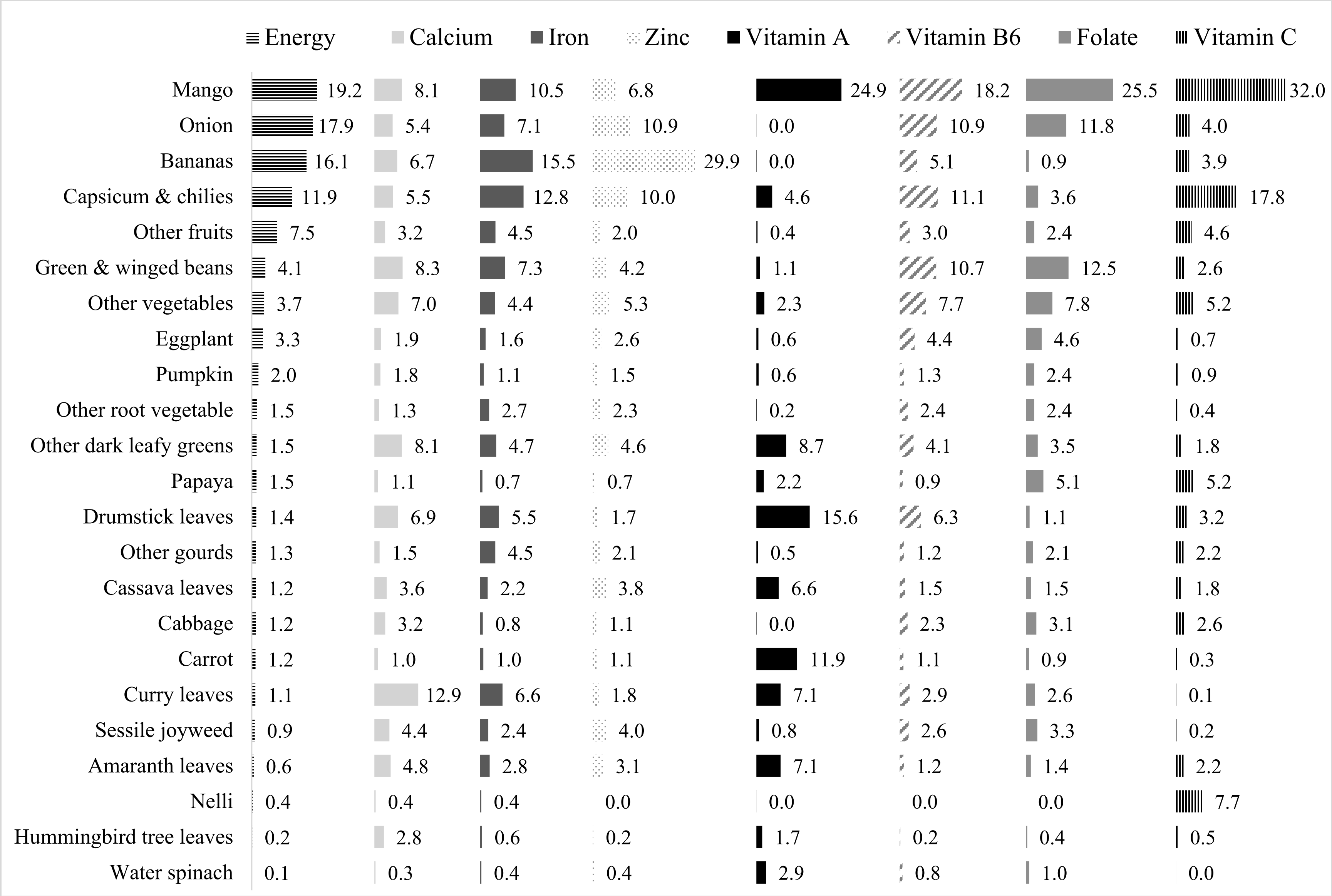

Supplemental Figure 1. Relative contribution of specific fruits and vegetables to total micronutrient intake from fruits and vegetables among Sri Lankan farming adults (% of total nutrients supplied by fruit & vegetables, N=1283)

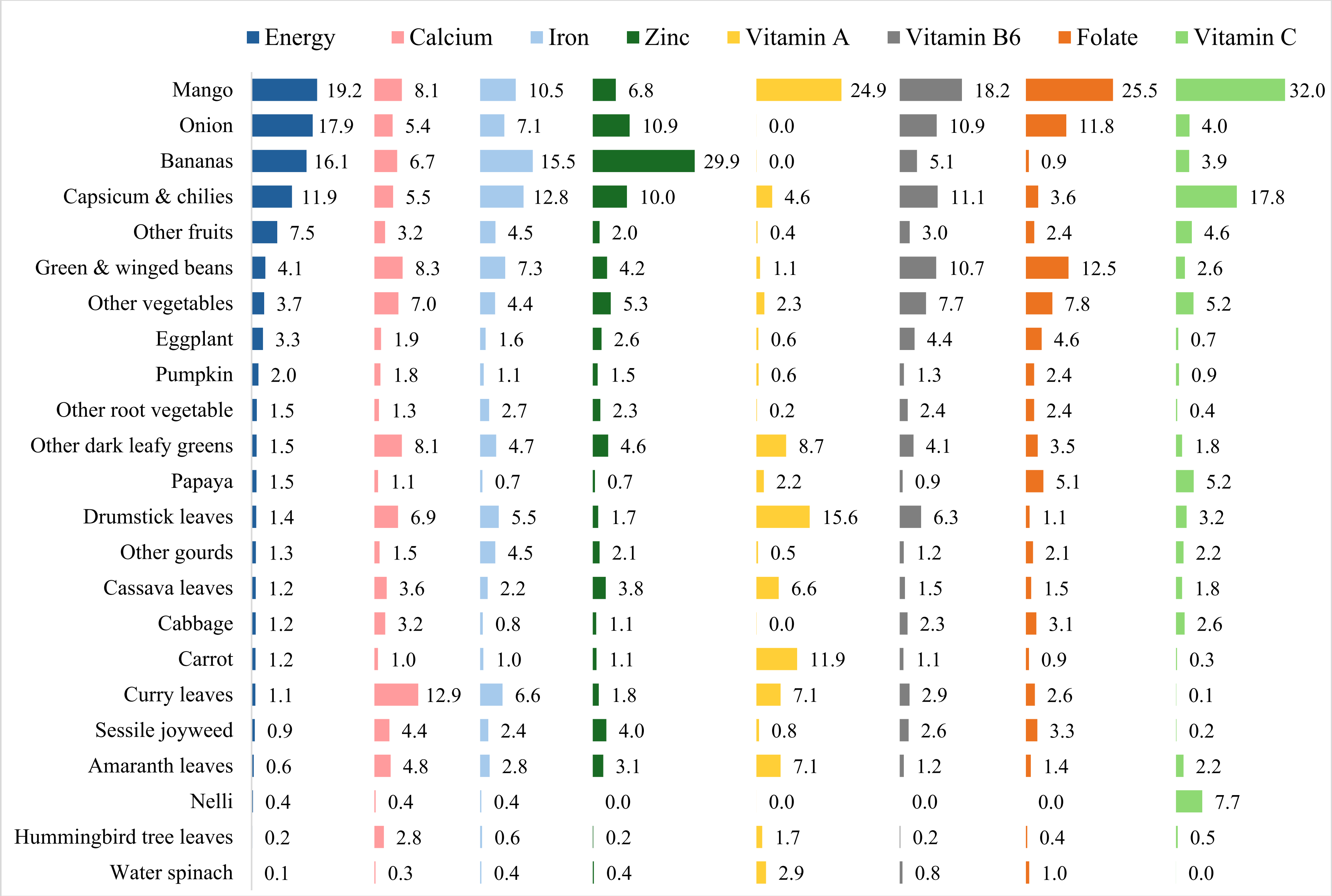

Supplement: Joyce et al. supplementary material [file S1368980025000072sup001.pdf]
